# A Single Amino Acid Substitution Changes Antigenicity of ORF2-Encoded Proteins of Hepatitis E Virus

**DOI:** 10.3390/ijms11082962

**Published:** 2010-08-12

**Authors:** Jiu-Hong Liang, Xing Dai, Chen Dong, Ji-Hong Meng

**Affiliations:** School of Medicine, Southeast University, Nanjing, Jiangsu 210009, China; E-Mails: liangjulia@163.com (J.-H.L.); daix60@yahoo.com.cn (X.D.); cdong1973@263.net (C.D.)

**Keywords:** hepatitis E virus, antigenicity, monoclonal antibody, amino acid mutation

## Abstract

Extensive genomic diversity has been observed among hepatitis E virus (HEV) strains. However, the implication of the genetic heterogeneity on HEV antigenic properties is uncertain. In this study, monoclonal antibodies (Mabs) against truncated ORF2-encoded proteins (aa452–617, designated p166 proteins) derived from HEV strains of Burma (genotype 1a, p166Bur), Pakistan (1b, p166Pak) and Morocco (1c, p166Mor) were raised and used for identification of HEV antigenic diversity. Six Mabs reacted to these 3 p166 proteins as well as p166 proteins constructed from strains derived from Mexico (genotype 2), US (genotype 3) and China (genotype 4), indicating the existence of pan-genotypic epitopes. Two Mabs, 1B5 and 6C7, reacted with p166Bur and p166Mor, but not p166Pak or p166s derived from genotypes 2, 3, and 4, indicating that these 2 Mabs recognized strain-specific HEV epitopes. Both the common and specific epitopes could not be mapped by 23 synthetic peptides spanning the p166Bur sequence, suggesting that they are confirmation-dependent. Comparative sequence analysis showed that p166Bur and p166Mor shared an identical aa sequence along their entire lengths, whereas for p166Pak the aas occupying positions 606 and 614 are different from aas at corresponding positions of p166Bur and p166Mor. Reactivity between 1B5 and p166Bur was abrogated with mutation of p166Bur/A606V, whereas p166Pak acquired the reactivity to 1B5 with mutation of p166Pak/V606A. However, mutations of p166Bur/L614M and P166Pak/M614L did not affect the immunoreactivity. Therefore, the aa occupying position 606 plays a critical role in maintaining the antigenicity of the HEV p166 proteins.

## 1. Introduction

Hepatitis E virus (HEV) is an enterically transmitted pathogen that causes epidemic and sporadic cases of hepatitis E predominantly in the developing countries of Asia and Africa. The mortality of the disease is high, up to 25%, in infected pregnant women. In industrialized countries, sporadic cases of hepatitis E have been reported, either imported by travelers from endemic regions or acquired indigenously [1]. Although usually presenting as an acute illness, chronic hepatitis E has been observed in recipients of solid-organ transplantation [2]. The diagnosis of the disease is largely dependent on the detection of anti-HEV antibodies by enzyme immunoassays such as enzyme-linked immunosorbent assay (ELISA)[3]. Vaccines are under development.

HEV was previously classified in the family *Calciviridae*, but it is now classified as being in the genus *Hepevirus* in the family *Hepeviridae* [4]. It is a non-enveloped virus with a single-stranded, positive-sense RNA genome of approximately 7.2 kb in length. The genome is composed of a 5′ untranslated region (UTR), three open reading frames (ORFs), a 3′ UTR, and a poly (A) tail. ORF1, ORF2 and ORF3 are partially overlapped and encode non-structural proteins, a structural capsid protein, and a small phosphoprotein, respectively. Although a single serotype has been proposed, extensive genomic diversity has been observed among HEV strains [5]. Based on the phylogenetic analysis of full genome sequences, HEV strains are classified into four major genotypes [6,7]. The representative prototypes of genotypes 1, 2, 3 and 4 are derived from the Burmese, Mexican, US and Chinese strains, respectively [8–11]. Sub-genotypes within each genotype are recognized [6,7]. However, the relationship between HEV genomic heterogeneity and HEV antigenic characters has not been comprehensively studied.

Commercial available HEV ELISA kits for anti-HEV detection are usually based on HEV genotype 1 and 2 antigens. Cross-reactivity among antigens obtained from different genotypes exists [12–15]. Nevertheless, Schlauder *et al.* [10] observed that although IgM class antibodies directed against HEV US-1 synthetic peptides were detected in a patient infected with HEV US-1, they could not be detected using synthetic peptides from the Burmese or Mexican strains of HEV. Various reports also indicate that the commercial assays based on HEV genotype 1 and 2 antigens have sometimes failed to detect antibodies in patients with proven HEV genotype 3 or 4 infections [16–19]. In accordance with these findings, our previous studies have identified a pan-genotype, conformation-dependent neutralization epitope in a 166-amino-acid segment of the ORF2 protein (p166) [20]. However, this segment also accommodates genotype-specific epitopes [21,22]. Similar results have been described by Schofield *et al.* [23] when they studied the antigenic sites of a 55 kD truncated ORF2 protein (aa112–607) expressed from baculovirus. The existence of antigenic heterogeneity between HEV genotypes appears to be an important factor to affect the accurate diagnosis of HEV infection. In the present study, monoclonal antibodies (Mabs) against p166 proteins from the Burmese, Pakistani and Moroccan strains, which belong to three subtypes of genotype 1 of HEV [24], were prepared to analyze antigenic heterogeneity among HEV sub-genotypes. As a result, a specific epitope, for the first time, was found exclusively in p166s of the Burma (p166Bur) and Morocco (p166Mor) strains, but not in p166 of the Pakistan strain (p166Pak) or other HEV strains belonging to genotypes 2, 3, and 4. Site-directed mutagenesis analysis indicated that a single amino acid (aa) change at position of 606 of the HEV ORF2-encoded protein resulted in the antigenicity change among different p166 proteins, especially between those of subtypes of genotype 1. This finding extends our knowledge on HEV heterogeneity and will help us to find a potential way to distinguish HEV stains, subtypes, or genotypes through a serologic tool in future.

## 2. Results

### 2.1. Preparation of Mabs against p166Bur, p166Pak and p166Mor

A total of 8 Mabs were obtained; 3 Mabs (1B5, 2C2 and 3G1) against p166Bur, 2 Mabs (3A3 and 5E9) against p166Pak, and 3 Mabs (6C7, 1A6 and 6G1) against p166Mor. All the Mabs were purified using immobilized protein G. Their concentrations ranged from 2.8 to 4.5 mg/mL.

### 2.2. Difference in Immunoreactivity of the Mabs

**Figure 1:**
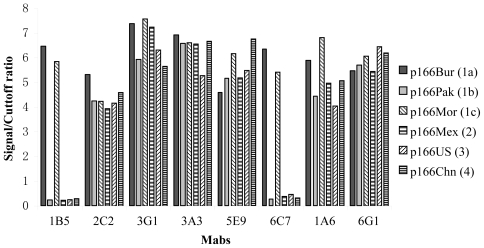
Immunoreactivity of the Mabs to p166 proteins derived from different HEV genotypes and subtypes. Bars indicated the results of the ELISA at Signal/Cutoff ratio. Signal/Cutoff ratio ≥1 was considered a positive result. Proteins used in the study were indicated right.

Mabs 2C2, 3G1, 3A3, 5E9, 1A6 and 6G1 cross-reacted with all of the p166 proteins (p166Bur, p166Pak, p166Mor, p166Mex, p166US and p166Chn), suggesting that these Mabs recognized epitopes common to all 4 HEV genotypes (Figure 1). However, Mabs 1B5 and 6C7 reacted only with p166Bur and p166Mor, but not p166Pak or p166 proteins derived from genotypes 2, 3 and 4 (Figure 1), suggesting recognition of an epitope specific and common to both p166Bur and p166Mor. p166Bur has been classified as belonging to subtype 1a, and p166Mor to 1c. They have an identical aa sequence within the region of p166. However, p166Pak, which is classified as subtype 1b, has 2 aas different from p166Bur and p166Mor: at positions 606 and 614 (Table 1).

### 2.3. Epitopes Recognized by the Mabs Are Conformation-Dependent

**Figure 2:**
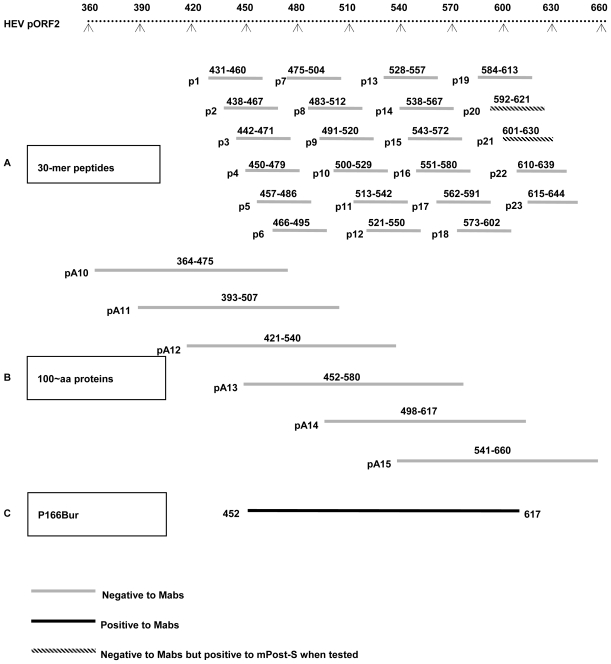
Characterization of epitopes recognized by the Mabs and modeled by different synthetic peptides and recombinant proteins. (**A**) Twenty-three overlapping 30-mer peptides tested by using ELISA. (**B**) Six 100-aa long recombinant proteins tested by using ELISA and Western blotting. (**C**) p166Bur tested by using ELISA and Western blotting.

Twenty-three 30-mer synthetic peptides (p1–23), spanning the sequence of p166Bur, were used for epitope mapping. Two C-terminal peptides (p20, spanning aa 592–621, and p21, spanning aa 601–630) showed ELISA reactivity with the p166 immune serum, mPost-S, but not with any of the Mabs. (Figure 2A). These data indicate that linear epitopes exist in p166 and are located within the C-terminus of p166. However, the epitopes recognized by the Mabs could not be modeled by any of the synthetic peptides, suggesting that they were conformation-dependent.

To further characterize these epitopes, 6 recombinant proteins (pA10–15), approximately 100 aa in length and covering the p166Bur aa sequence, were used as antigens. None of them showed any reactivity by ELISA or Western blotting to any of the Mabs (Figure 2B). However, while the p166Bur was used as antigen, all the Mabs were reactive to it (Figure 2C). Compared to p166Bur spanning aa452–617, pA13 (spanning aa452–580) and pA14 (spanning aa499–617) were shorter by 37 aa at the C-terminus and 46 aa at the N-terminus, respectively. The lack of immunoreactivity of the two shorter proteins to the Mabs indicates that the N-terminal and C-terminal residues of p166 are required to maintain the conformation of the epitopes.

### 2.4. Position 606 Is Critical for the Epitope Recognized by Mab 1B5 and 6C7

**Figure 3:**
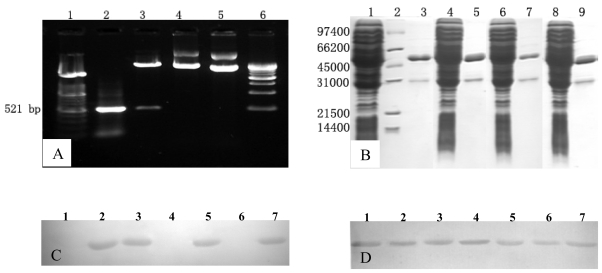
**Analysis of Mab 1B5 against p166 mutants with single amino acid mutation.** (**A**) Site-directed mutagenesis construction. Lane 1: DNA molecular weight marker XIV (100 base-pair ladder, Roche Molecular Biochemicals). Lane 2: p166Bur/A606V PCR product. Lane 3: digestive products of pGEX-4T-2-p166Bur/A606V with BamHI and XhoI. Lane 4: pGEX-4T-2-p166Bur/A606V. Lane 5: pGEX-4T-2. Lane 6: DNA molecular weight marker XVII (500 base-pair ladder, Roche Molecular Biochemicals). (**B**) Four p166 mutants expressed in *Escherichia coli* and separated by SDS-PAGE. Lanes 1 and 3: lysis mixture and purified protein of p166Pak/M614L. Lane 2: molecular weight markers (low range, Bio-Rad, Hercules, CA). Lane 4 and 5: lysis mixture and purified protein of p166Pak/V606A. Lane 6 and 7: lysis mixture and purified protein of p166Bur/A606V. Lane 8 and 9: lysis mixture and purified protein of p166Bur/L614M. (**C**) Western blotting analysis of Mab 1B5 against the p166 mutants. Lane 1–7: purified p166Pak/M614L, p166Pak/V606A, p166Bur/L614M, p166Bur/A606V, p166Bur, p166Pak, and p166Mor (**D**) Western blotting analysis of Mab 3G1 against the p166 mutants. Lane 1–7: same as (C).

**Figure 4:**
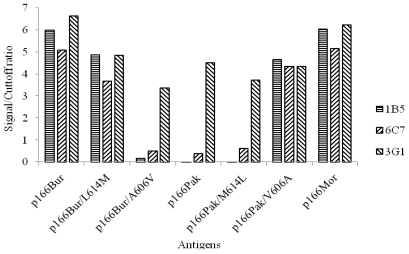
Immunoreactivity of the Mabs to p166 mutants with single amino acid change. Bars indicate the results of the ELISA at Signal/Cutoff ratio. Signal/Cutoff ratio ≥1 was considered a positive result. Proteins used in the study are indicated below.

To determine if the aa residues located at aa 606 and aa 614 play a role in determining the specificity of Mabs 1B5 and 6C7 to p166Bur and p166Mor but not of p166Pak, a primer-directed mutagenesis was utilized to express p166 with mutations at these two positions. Four p166 mutants, p166Pak/V606A, p166Pak/M614L, p166Bur/A606V and p166Bur/L614M, were constructed and expressed in *E. coli* (Figure 3A, B). Western blotting analysis showed that Mab 1B5 and 6C7 reacted with p166Pak/V606A and p166Bur/L614M, but not with p166Pak/M614L and p166Bur/A606V (Figure 3C). Mab 3G1 reacted well with all the p166 mutants (Figure 3D). Similar results were observed by ELISA (Figure 4). Thus, reactivity of Mab 1B5 and 6C7 with p166Bur was abrogated when aa606 was changed from A to V, whereas the reactivity was not affected when position aa614 was changed from L to M. However, p166Pak became immunoreactive with Mab 1B5 and 6C7 when position aa606 was changed from V to A. No reactivity was observed between Mabs 1B5 and 6C7 with p166Pak, even when position aa614 was changed from M to L (Figures 3C and 4). Consequently, position aa606 is critical for the epitope recognized by 1B5 and 6C7.

## 3. Discussion

Significant epidemiological differences have been observed among HEV genotypes. HEV genotypes 1 and 2 are associated with infection and disease in humans and experimentally infected nonhuman primates, not pigs. These two genotypes circulate primarily in tropical and subtropical developing countries of Asia and Africa and are responsible for hepatitis E epidemics there. By contrast, HEV genotypes 3 and 4 appear to circulate more widely and have been identified to infect humans as well as pigs [25–29], boars [30–33] and other mammals including deer [31,34] and rabbits [35]. Humans can acquire infection by these genotypes via ingestion of undercooked boar meat and venison [34,36]. Moreover, it was shown that diagnostic assays based on peptides derived from genotypes 1 and 2 may fail to detect HEV infection caused by genotypes 3 or 4 [10,11], suggesting genotype-specific antigenic differences.

The p166 protein spanning the C-terminal segment of the HEV pORF2 (aa452–617) was previously determined to contain HEV neutralization epitope and the most immunogenic site on pORF2 with a conformation-dependent character [13,20,37]. It is highly susceptible to renaturation after the SDS-PAGE and capable of interacting with corresponding antibodies in Western blotting [20,38]. In the present study, recombinant p166 proteins derived from three subtypes of genotype 1 were used as immunogens to raise murine Mabs. Six Mabs showed cross-reactivity with p166 proteins generated from all 4 genotypes of HEV, indicating that common epitopes exist in different HEV genotypes. However, two Mabs, 1B5 and 6C7, showed specific reactivity to p166Bur and p166Mor but not to p166Pak or p166 proteins derived from genotypes 2, 3, 4. This set of results indicated that some specific epitopes exist within p166s. Sequence analysis revealed that p166Bur has an aa sequence identical to p166Mor in spite of a low homology of 88.6% in nucleotide sequences between them. Thus, both proteins should have the same antigenic epitope recognized by Mabs 1B5 and 6C7. Since this epitope could not be mapped with the 23 overlapping, linear peptides spanning the p166 sequence, it is likely to be conformation-dependent. Furthermore, neither the recombinant protein pA13 that spans between aa 452 to 580, nor the recombinant protein pA14 that spans between aa 498 to 617, reacted with Mabs 1B5 and 6C7, suggesting that the N-terminal 46 (aa452–497) and the C-terminal 37 (aa581–617) residues of p166 are essential to maintain the epitope conformation. Although p166Pak shares even closer nucleotide-sequence homology (93%) with p166Bur than that with p166Bur and p166Mor (88.6%), sequence analysis showed that p166Mor shares the same aa sequence with p166Bur, whereas p166Pak has two aas different from p166Bur and p166Mor, at positions 606 and 614. These positions are, respectively, occupied by A and L in p166Bur/p166Mor, and V and M in p166Pak. It is notable that aa614 is most variable among the HEV sequences deposited in GenBank, and this variability is genotype-associated. Aa614 is occupied by L in genotype 1a and 1c, M in 1b, F in genotype 2, and A or V in genotypes 3 and 4 (see Table 1). By contrast, the aa occupying position 606 is not genotype-specific. Mutation analysis reveals that changes at position 614 did not affect the reactivity of Mab 1B5 and 6C7 against p166Bur and p166Pak. Position 606 was found to be critical for the reactivity of the epitope recognized by 1B5 and 6C7, despite the lesser variability at this position.

Recently, certain novel biological and immunological characteristics of HEV-like particles have been identified based on the crystal structure [39]. The truncated HEV capsid protein was found to comprise 3 domains, designated S (shell), M (middle) and P (protruding), derived from aa 129–319, 320–455 and 456–606, respectively. Position 606 is the last aa residue in the P domain, which is involved binding to host cells susceptible to HEV infection and has some neutralization epitopes. Among HEV sequences available in GenBank, only 10 have position 606 occupied by V, including two Pakistani strains (Sar-55 and 87-Pakistan-B)[40], two HEV plasmids (pSK-HEV-2 and pSK-HEV-3) derived from Sar-55, two Japanese strains (E087-SAP04C and E067-SIJ05C) derived from patients who had traveled to Shanghai (China), and three swine HEV isolates (swDQ1, Ch-S-1, and SH SW-zs1 [41]). However, they are not available for us to test with Mabs 1B5 and 6C7. Recently, a Chinese HEV strain, W01, was sequenced in our lab and was identified to have the same aa occupying position 606 as the Burma-82 strain, but its position 614 was occupied by M as the Pakistani Sar-55 strain. The p166 based on W01 was constructed and found to react well to Mabs 1B5 and 3G1 (data not shown), indicating the important role of the position of aa606 in the reactivity.

Single aa changes have been observed to alter the antigenicity, virulence, or both of a virus, e.g., the 1918 influenza virus [42] and poliovirus [43]. It has been speculated that specific aas might be responsible for influencing transmission route, host specificity, geographic limitations, genetic variance, and immunological escape. Several mutations have been noted in the region at positions 452–617 of ORF2 of HEV despite the high conservation of this region. These mutations are broadly genotype-specific (Table 1). Antigenic analysis with Mabs may provide a means for determining aas that contribute to specific epitopes [23]. Genotyping is onerous as it is dependent on nucleotide sequencing. Serotyping would be a more convenient tool of use in epidemiological studies. The data generated from the present study suggest that a serological approach to discrimination of HEV strains may be feasible. Further investigations into the antigenic specificity of HEV are warranted.

## 4. Materials and Methods

### 4.1. Recombinant Proteins and Synthetic Peptides

The HEV p166 recombinant plasmids were constructed by inserting the PCR fragments encoding aa 452–617 of the ORF2 protein of the HEV Burma-82 strain (genotype 1a), the Pakistani Sar-55 strain (genotype 1b), the Morocco strain (genotype 1c), the Mex-14 strain (genotype 2), the US-1 strain (genotype 3) and the Chinese CN9829 strain (genotype 4) (GenBank accession numbers are, respectively, M73218, M80581, AY23020, M74506, AF060668 and AY789225) into the pGEX-4T-2 vector (Pharmacia Biotech Inc., Piscataway, NJ). The primers used to amplify the PCR fragments are listed in Table 2. The plasmid was then used to transform competent *E. coli* JM109 cells. After the inserted sequence was confirmed by DNA sequencing, the expression was induced by isopropyl β-D-thiogalactoside. The expressed soluble p166 was thereafter purified using the Bulk and Redipack GST Purification Modules system (Pharmacia) according to the manufacturer’s instruction. The resultant p166 recombinant proteins are designated p166Bur, p166Pak, p166Mor, p166Mex, p166US, and p166Chn, respectively, as described previously [44].

Four mutant proteins containing a single aa change at position 606 or 614 of the p166Bur or p166Pak were generated using a PCR strategy for site-directed mutagenesis and the plasmid of p166Bur or p166Pak as PCR template. They, p166Bur/A606V, p166Bur/L614M, p166Pak/V606A, and p166Pak/M614L, were expressed in *E. coli* JM109 and purified as described above. The primers used in site-directed mutagenesis are shown in Table 2.

Six approximately 100 aa long and overlapping proteins, pA10, pA11, pA12, pA13, pA14 and pA15, spanning the aa 364–660 region of the Burmese pORF2 were expressed as previously described [20]. The primers used to amplify the corresponding sequences are shown in Table 2. Additionally, 23 overlapping 30-mer peptides, p1 to p23, that encompass aa 431–644 of ORF2 of the HEV Burmese strain synthesized as previously described [45] were used in this study. The specific location of the proteins and the peptides are shown in Figure 2.

### 4.2. Generation of Mabs

Three groups of 8-week-old female BALB/c mice were immunized 3 times with equal volumes (40 μg in 40 μL) of p166Bur, p166Pak or p166Mor, respectively. Mouse spleen cells were fused with sp2/0 mouse myeloma cells using polyethylene glycol 1500 (50% wt/vol) as previously described [46]. Mab screening was conducted by an ELISA using the immunizing antigens. Since the antigens are GST-fusion proteins, purified GST was used for a parallel test. Only the hybridomas positive to the antigen but negative to GST were further sub-cloned 3 times by limitation dilution. The hybridoma cells secreting specific antibodies against p166Bur, p166Pak or p166Mor were harvested and inoculated intraperitoneally to BALB/c mice. Ascitic fluid was collected 7 to 10 days later and filtered after centrifugation, purified with a protein G affinity column, and then stored at –70 °C until further use.

### 4.3. ELISA

Microtiter plates were coated with each of the 100-aa truncated proteins, 30-mer peptides at a concentration of 5 μg mL^−1^, or p166 proteins and p166 mutants at a concentration of 1 μg mL^−1^. Purified Mabs diluted 1:100 were added in the coated wells. Mouse pre-immune (mPre-S) and post-immune (mPost-S) sera obtained from the mice immunized with the p166 proteins were used as controls. ELISA was carried out as previously described [47]. The cutoff value was set at ≥0.2 of *OD_450_*.

### 4.4. Western Blotting

HEV proteins were heated in 2X loading buffer and electrophoresed in a 12% SDS-PAGE. Electrophoretic transfer of the proteins to a nitrocellulose membrane was carried out at 50 mA for 2 h at 4 °C. The membrane was blocked for 1 h with 5% skim milk in PBS and then incubated overnight at 4 °C with supernatant from the hybridoma cell culture or a 1:100 dilution of mPre-S or mPost-S. After six washes of 10 min each, a horseradish peroxidase-conjugated anti-mouse IgG (KPL, Gaithersburg, MD, USA) was added at a dilution of 1:2,000 in 5% skim milk in PBS. After 1 h of incubation, the blots were washed and 3, 3′-diaminobenzidine (DAB) was added to visualize.

## Figures and Tables

**Table 1:** Mutations associated with HEV genotypes/subtypes within the p166 region.

HEV genotype		Mutations associated with HEV genotypes/subtypes within the p166 region*																			
		483	490	492	497	500	509	511	517	527	537	569	571	580	587	590	593	599	606	609	614
	1a	S	G	V	S	L	A	A	T	S	F	L	V	A	S	A	V	A	A	L	L
**1**	1b	S	G	V	S	L	A	A	T	S	F	L	V	A	S	A	V	A	A/V	L	M
	1c	S	G	V	S	L	A	A	T	S	F	L	V	A	S	A	V	A	A	L	L
	**2**	S	G	V	S	L	A	A	S	P	F	I	I	A	R	A	V	A	A	L	F
	**3**	T	N	M	T	F/L	A	A	S	T	Y	I	I/V	A	S	A	T	G	A/V	V	A/V
	**4**	T	N	M	T	F	G	S	S	T/M	F/Y	I	I	C	N	S	V	G	A	A/V	V/A

* Eighty-two full length HEV sequences were involved in the analysis. Frequency of amino acid mutation identified in less than 3 HEV sequences was not reckoned in.

**Table 2:** Primers used for construction of recombinant GST-fusion HEV ORF2 proteins.

Proteins	Primer sequence
pA10 (aa 364–475)	Forward 5′-CCC*GGATCC*ATCGGCCGCGGGATAGCCCTCAC-3′ Reverse 5′-CCC*CTCGAG*TCAGAGAGAGAGCCAAAGCACATC-3′
	
pA11 (aa 393–507)	Forward 5′-CCC*GGATCC*GGCCAGCTGTTCTACTCCCGTCC-3′ Reverse 5′-CCC*CTCGAG*TCACGCGCCGGTCGCAACATTAACC-3′
	
pA12 (aa 421–540)	Forward 5′-CCC*GGATCC*CAGGATAAGGGTATTGCAATCC-3′ Reverse 5′-CCC*CTCGAG*TCACGGCAGGACAAAGAAGGTCTTC-3′
	
pA13 (aa 452–580)	Forward 5′-CCC*GGATCC*CCGACGCCTTCTCCAGCCCCATC-3′ Reverse 5′-CCC*CTCGAG*TCAAGCGACCCGGTGCCCGGCGGCA-3′
	
pA14 (aa 498–617)	Forward 5′-CCC*GGATCC*ACCTTGGTTAATGTTGCGACC-3′ Reverse 5′-CCC*CTCGAG*TCAAGGGTAGTCCAAGGTATCCTCAA-3′
	
pA15 (aa 541–660)	Forward 5′-CCC*GGATCC*CTCCGCGGTAAGCTCTCTTTC-3′ Reverse 5′-CCC*CTCGAG*CTACAACTCCCGAGTTTTACC-3′
	
p166Bur (aa 452–617)	Forward 5′-CCC*GGATCC*CCGACGCCTTCTCCAGCCCCATC-3′ Reverse 5′-CCC*CTCGAG*TCAAGGGTAGTCCAAGGTATCCTCAA-3′
	
p166Bur/A155V	Mutant primer 5′-CTCTGTGCTAGCATTGCTTGAGGATAC-3′ Forward 5′-CCC*GGATCC*CCGACGCCTTCTCCAGCCCCATC-3′ Reverse 5′-CCC*CTCGAG*TCAAGGGTAGTCCAAGGTATCCTCAA-3′
	
p166Bur/L163M	Forward 5′-CCC*GGATCC*CCGACGCCTTCTCCAGCCCCATC-3′ Reverse 5′-CCC*CTCGAGT*CAAGGGTAGTCCATGGTATCCTCAA-3′
	
p166Pak (aa 452–617)	Forward 5′-CCC*GGATCC*CCGACACCTTCCCCAGCCCCATC-3′ Reverse 5′-CCC*CTCGAG*TCAAGGGTAGTCCATGGTATCCTCAA-3′
	
p166Pak/V155A	Mutant primer 5′-CTCTGCGCTAGCATTGCTTGAGGATAC-3′ Forward 5′-CCC*GGATCC*CCGACACCTTCCCCAGCCCCATC-3′ Reverse 5′-CCC*CTCGAG*TCAAGGGTAGTCCATGGTATCCTCAA-3′
	
p166Pak/M163L	Forward 5′-CCC*GGATCC*CCGACACCTTCCCCAGCCCCATC-3′ Reverse 5′-CCC*CTCGAG*TCAAGGGTAGTCCAAGGTATCCTCAA-3′
	
p166Mor (aa 452–617)	Forward 5′-CCC*GGATCC*CCGACACCTTCCCCAGCCCCGTC-3′ Reverse 5′-CCC*CTCGAG*CCCACCCCGTCGCCTGCGCCATC-3′
	
p166Mex (aa 452–617)	Forward 5′-CCC*GGATCC*CCCACCCCGTCGCCTGCGCCATC-3′ Reverse 5′-CCC*CTCGAG*TCACGGATAATCAAAAGTATCCTCCA-3′
	
p166US (aa 452–617)	Forward 5′-CCC*GGATCC*CCTACCCCGTCACCTGCCCCCTC-3′ Reverse 5′-CCC*CTCGAG*TCAAGGATAATCAACAGTATCCTCGA-3′
	
p166Chn (aa 452–617)	Forward 5′-CCC*GGATCC*CCTACCCCCTCTCCTGCTCCCTC-3′ Reverse 5′-CCC*CTCGAG*TCAAGGGTAATCAACAGTGTCCTCCA-3′
